# A Novel Multiplex RT-PCR Assay for Simultaneous Detection of Dengue and Chikungunya Viruses

**DOI:** 10.3390/ijms21218281

**Published:** 2020-11-05

**Authors:** Mohammed Alimul Islam, Mohamed E. El Zowalaty, Sumaiya Islam, Mohiuddin Sharif, Md. Rajibur Rahman, Mohammad Robed Amin, Md. Mortuza Ali, Md. Tanvir Rahman, Kouichi Morita, Hossam M. Ashour

**Affiliations:** 1 Department of Microbiology and Hygiene, Faculty of Veterinary Science, Bangladesh Agricultural University, Mymensingh 2202, Bangladesh; tanvirahman@bau.edu.bd; 2 Department of Clinical Sciences, College of Medicine, University of Sharjah, Sharjah 27272, UAE; 3 Zoonosis Science Center, Department of Medical Biochemistry and Microbiology, Uppsala University, SE 75123 Uppsala, Sweden; 4 Department of Medicine, Bangladesh Medical College and Hospital, Dhaka 1209, Bangladesh; yuki.islam@icloud.com; 5 Department of Medicine, Dhaka Medical College and Hospital, Dhaka 1000, Bangladesh; mohiuddinsharif.fmc@gmail.com (M.S.); dr.rajiburrahman@gmail.com (M.R.R.); robedamin@yahoo.com (M.R.A.); 6 Department of Community Medicine, Monno Medical College and Hospital, Manikganj 1800, Bangladesh; dr.mortuza@gmail.com; 7 Department of Virology, Institute of Tropical Medicine, Nagasaki University, 1-12-4, Sakamoto, Nagasaki 852-8523, Japan; moritak@nagasaki-u.ac.jp; 8 Department of Integrative Biology, College of Arts and Sciences, University of South Florida, St. Petersburg, FL 33701, USA; 9 Department of Microbiology and Immunology, Faculty of Pharmacy, Cairo University, Cairo 11562, Egypt

**Keywords:** dengue virus, chikungunya virus, arboviruses, Flaviviridae, Togaviridae, mRT-PCR, sensitivity, specificity

## Abstract

The goal of the study was to develop a specific, sensitive, and cost-effective molecular RT-PCR diagnostic assay for the rapid and simultaneous detection of the serotypes of dengue virus (DENV) and Chikungunya virus (CHIKV) from sera of suspected febrile patients. A single-tube, single-step multiplex RT-PCR (mRT-PCR) assay was designed for the detection of viral genomes from clinical and field samples. Specificity and sensitivity of the mRT-PCR assay were evaluated against six different combinations using two reverse transcriptases (AMV-RT and RT-Ace) and three DNA polymerases (LA-Taq, rTaq, and Tth). Among the six combinations, the AMV-RT and LA-*Taq* combination was more specific and sensitive than other enzyme combinations for detecting viral genomes of DENV-1, DENV-2, DENV-3, and DENV-4 (*p* < 0.01), and for detecting viral genomes of CHIKV (*p* < 0.05). The detection limits of the mRT-PCR were 10 focus forming units (FFU) for CHIKV and 1 FFU, 20 FFU, 0.1 FFU, and 10 FFU for DENV-1, DENV-2, DENV-3, and DENV-4, respectively. The primers used for the mRT-PCR did not show any cross-reactivity among the serotypes of DENV or CHIKV. Specificity and sensitivity of the newly developed mRT-PCR were validated using serum samples collected from febrile patients during dengue outbreaks in Bangladesh. The sensitivity for serotype detection of DENV and CHIKV was superior to the virus isolation method and the antigen detection method using the Dengue NS1-Ag assay. This novel mRT-PCR method can be used for molecular epidemiological surveillance of DENV and CHIKV in epidemic and endemic countries.

## 1. Introduction

Dengue virus (DENV) and Chikungunya virus (CHIKV) are arboviruses of the family *Flaviviridae* and *Togaviridae* respectively. *Aedes* mosquitoes such as *Aedes aegypti* and *Aedes albopictus* play major roles in the transmission of these viruses to humans and animals. Simultaneous infections with both viruses have been reported and can increase the case fatality rates. Humans in tropical and subtropical areas are at a high risk of infection caused by these viruses. There are four medically important disease-causing serotypes of DENV (DENV-1, DENV-2, DENV-3, and DENV-4) [1,2]. These serotypes can infect individuals of different age groups in tropical and sub-tropical countries [1,2]. On the other hand, CHIKV has a single serotype and is distributed in Africa, Asia, Australia, and North America [1,2]. For the control and prevention of dengue and chikungunya viral fever, it is important to confirm the serotypes of the circulating viruses. Dengue is the tenth leading cause of death in the world [3]. DENV is an enveloped single-stranded positive-sense RNA virus. It is estimated that billions of humans are at risk of DENV infections [4,5,6]. It can weaken the immune system causing people to be vulnerable to other emerging diseases such as encephalitis [7]. CHIKV is a positive-sense RNA virus that is transmitted by the same mosquito vectors of DENV, and causes fever with similar symptoms to the classical dengue fever [8]. After its first appearance in the Newala district in Tanzania in 1953, CHIKV was transmitted to other countries [9].

In recent years, diseases such as self-limiting febrile illness and hemorrhagic fever have been reported in many tropical and sub-tropical countries [10]. When the multiple serotypes of DENV circulate concurrently with CHIKV, there is a higher risk for more severe forms of the disease such as dengue hemorrhagic fever (DHF) and dengue shock syndrome (DSS) [11,12,13,14]. This highlights the importance of the accurate determination of the circulating viral serotypes at any given location [15,16].

Diagnostic methods commonly used for viral diseases of the family *Flaviviridae* and *Togaviridae* include virus-specific antibody detection, virus isolation and characterization, and viral genome detection using nucleic acid amplification methods such as RT-PCR [17,18,19,20,21]. Serological methods for diagnosis of viruses belonging to these families include hemagglutination inhibition (HI), serum neutralization test (SNT), plaque reduction neutralization test (PRNT),**** Immunofluorescence assay (IFA), and capture immunoglobulin G (IgG)/immunoglobulin M (IgM) enzyme-linked immunosorbent assay (ELISA) [22,23,24,25,26]. IgM and IgG ELISA tests are widely used for rapid serological diagnosis, but have the limitation of the inability to identify the circulating viral serotypes [26]. Viruses responsible for encephalitis in the family *Flaviviridae* cannot be differentiated serologically using the cross-antibody test [24,27]. The IgG antibody has a high degree of cross-reactivity between homologous and heterologous antigens of viruses of the *Flaviviridae* and *Togaviridae* families [24]. Serodiagnosis of past and present DENV and CHIKV infections is difficult due to the long persistence of IgG antibodies. Moreover, patients may have multiple and sequential co-infections or superinfections with DENV and CHIKV due to the lack of cross-protective neutralizing antibodies against these viruses [24].

Despite limitations such as lower sensitivity and longer duration, virus isolation using cell culture and infection of the mosquitoes are still commonly used for the detection and identification of DENV and CHIKV [28,29,30]. In order to improve sensitivity and reduce time for identification of viruses in infected cells and culture fluids, RT-PCR assays can be combined with cell culture [31,32,33]. The field of molecular diagnostics has been transformed over the past decade, leading to more reliable assays for the detection and characterization of viral pathogens. This is notable given the lack of reliable and sensitive assays for the detection of viruses from acute-phase serum, cerebrospinal fluid (CSF), infected culture fluid (ICF), and mosquito vectors [34,35].

Various molecular methods such as two-step RT-PCR, nested RT-PCR, quantitative real-time RT-PCR (qRT-PCR), and nucleic acid sequence-based amplification (NASBA) have been used for the identification of viruses [33,36,37,38,39,40,41]. These molecular methods have gradually become the preferred standard methods over virus isolation and sero-confirmation for the detection of viruses in acute-phase patient samples.

A single-tube, single-step multiplex RT-PCR (mRT-PCR) that is sensitive, specific, and rapid could offer a more cost-effective alternative to currently available methods for the detection of the serotypes of DENV and CHIKV [36,42]. Early viral detection and rapid viral serotyping from patient serum samples can provide important information for epidemiological studies of different outbreaks [43]. Moreover, the detection of DENV and CHIKV in mosquitoes is also useful for the determination of the prevalence of diseases and the nature of the viral vectors [36]. It is noteworthy that nucleic acid-based amplification methods have several intrinsic disadvantages such as the requirement of a highly precise equipment for amplification and elaborate, complicated methods for the detection of the amplified products. To reduce time and minimize complications, the two-step RT-PCR approach was later modified into a single-step mRT-PCR for the detection and serotyping of dengue. However, this mRT-PCR method was only found to be less sensitive [36,42,44].

The present study was designed to develop a single-tube, single-step mRT-PCR for the rapid and simultaneous detection and serotyping of DENV and CHIKV from clinical and mosquito samples. The sensitivity and specificity of the mRT-PCR assay were determined. We also assessed the applicability and feasibility of the assay for the confirmatory diagnosis of DENV and CHIKV viral fever.

## 2. Materials and Methods

### 2.1. Ethics

The study was approved by the ethical review committee of Dhaka Medical College (DMC), Dhaka, Bangladesh (Reference number: DMC/Research-16/2016/74).

### 2.2. Reference Virus Strains, Enzymes, and Primers

All four serotypes of dengue virus (DENV); dengue virus serotype-1 (DENV-1/Thai and DENV-1/Hawaii), dengue virus serotype-2 (DENV-2 ThNH-7/93, DENV-2 SL-04-111, and DENV-2 002ST-22A), dengue virus serotype-3 (DENV-3 BDH02-02, DENV-3 SL-03-11701, and DENV-3 SLMC-50), dengue virus serotype-4 (DENV-4 SL-04-8532, DENV-4 SLMC-318, and DENV-4), and chikungunya viruses (CHIKV Strain S27-African prototype and CHIKV M29-98, CHIKV M16-98) were obtained from the Department of Virology, Institute of Tropical Medicine, Nagasaki, Japan.

Six enzyme combinations of two reverse transcriptases (AMV-RT and RT-Ace) and three DNA polymerases (LA-Taq, r-Taq, and Tth) were used in this study.

Primers for the newly developed mRT-PCR included one sense (DC-S) and four complementary (D1-C, D2-C, D3-C, and D4-C) primers for the four DENV serotypes in addition to one sense (CHIKV-S) and one complementary (CHIKV-C) primers for the CHIKV.

### 2.3. Design and Selection of Primers for the mRT-PCR

Type-specific oligonucleotide sense (dengue consensus sense, DCS) and complementary primers against DENV-1, DENV-2, DENV-3 and DENV-4, and CHIKV sense and complementary primers were used for the mRT-PCR for DNA amplification of each serotype of DENV and CHIKV. These were designed based on the sequences of the C, PrM, M, and E1 genes of the known serotypes of DENV and CHIKV [36]. The list of primers is shown in Table 1. All the general criteria for primer design were followed including minimizing dimer formation and hairpins. The specificity testing for primers was carried out for the positive control of all four serotypes of DENV and for the single serotype of CHIKV.

Nucleotide sequences of the prototype strains of DENV and CHIKV were downloaded from the GenBank (accession numbers are shown in Table 1) and were aligned with the available sequences of other strains of each virus to identify homology using DNASIS software (Hitachi, Japan).

The primer sets used for the detection of the four serotypes of DENV and CHIKV were also cross-checked with the sequences of each serotype of DENV and CHIKV using Oligo Primer Analysis Software version 7. Moreover, each primer was also checked for their melting temperature, internal stability, primer-dimer and hairpin formation, and AT:GC ratio (data not shown).

The primer concentrations for the uniplex RT- PCR (uRT-PCR) and mRT-PCR were reduced many folds compared to the concentrations used in the two-enzyme assay protocol described by Kumaria and Chakravarti, [43] and no co-solvents were used. RNA extracts from the four known serotypes of DENV and CHIKV were used as positive controls.

### 2.4. Selection of Reverse Transcriptase and DNA Polymerase for mRT-PCR

Six combinations of two enzymes were made using two reverse transcriptase, Avian Myeloblastosis Virus (AMV) Reverse Transcriptase (AMV-RT and RT-ACE), and three DNA polymerase (*LA-Taq, r-Taq, Tth*) enzymes (Table 2).

In Table 2, the presence of an identical letter indicates the lack of any statistically significant difference, whereas the presence of different letters (For example, a versus b) indicates a significant difference as per Duncan’s Multiple Range Test (DMRT).

### 2.5. Patient Sera

A total of 650 blood samples (1 mL) were collected using non-heparinized vacutainer from dengue and chikungunya suspected acute-phase (day 1–6 of fever) febrile patients admitted in different hospitals of Dhaka city, Bangladesh during the dengue outbreaks in 2016, 2017, and 2018. Blood samples of febrile patients were collected randomly (either NS1 positive or negative) and were transported to the laboratory for serum preparation. Sera from blood of the DENV and CHIKV suspected patients were collected aseptically and used for serotype detection and virus isolation. For further analysis, the remaining sera were stored at −80 °C.

### 2.6. Virus Isolation, Stock Preparations, and Focus Assay

C6/36 cells from *A. albopictus* was used for the isolation of DENV and CHIKV from serum samples as previously described [31]. In brief, twenty clones were developed by culturing *A. albopictus* (Singh) cells in the presence of anti-CHIKV serum. Each clone of cells was tested for its yield of DENV 1, 2, 3 and 4 serotypes and also CHIKV. Of the twenty clone, clone 6 showed the highest yield of virus isolation. Presence of viruses in the ICF was verified by Ag-Capture ELISA and mRT-PCR.

The focus-forming assay for different serotypes of DENV was performed using the BHK-21 cell line as previously described [45]. BHK-21 cells were grown in 24-well plates. The confluent monolayer of the 24-h cultured cells was infected with different serotypes of DENV (0.1 mL/well of ICF of different dilutions from 10-1 to 10-10). After 1 h of incubation at 37 °C, the wells were washed three times with sterile PBS and then 0.5 mL of maintenance media was added (MEM with 1% FBS)/well and incubated for 24 h. An overlay of each well was then done with 1% methyl cellulose plus two times strong MEM and 2% FCS. After 72 h of infection, the methyl cellulose was discarded from each well and the cells were fixed with 4% para-formaldehyde for 1 h. Each well was washed three times with PBS. 1% Nonidet P-40 (non-ionic detergent) of 0.2 mL was added to each well and incubated at room temperature for 30 min. The wells were washed three times with PBS and then each well was blocked with 0.2 mL/well of 4% blockage solution (4g of V fraction of bovine serum albumin) and were incubated at room temperature for 1 h then the plates were washed three times with PBS. After washing, two-fold-diluted 0.2 mL/well of primary antibody (7E8) was added to each well and the plate was incubated at 37 °C for 1 h and then washed three times with PBS. 0.2 mL/well of conjugate (anti-mouse MAb at a dilution of 1:1000) was added to each well and incubated for 1 h and then washed three times with PBS. Finally, 0.2 mL of the substrate; one tablet of 3,3′-Diaminobenzidine (DAB) dissolved in 12 mL of PBS and 12 µl of 30% hydrogen peroxide; was added. The reaction was stopped with 1N sulphuric acid and then observed under fluorescent microscope for the detection of the FFU of the DENV in the infected cells.

### 2.7. Extraction and Preparation of Spiked Viral RNA with Mosquito’s Tissue Homogenate

Viral RNA was extracted from 70–140 µL serum obtained from patients whose viral titres were not known and also from the infected culture fluid (ICF) and mosquito tissue homogenate (MTH) spiked with RNA of all four serotypes of DENV and CHIKV of known focus forming unit (FFU) (100, 50, 20, 10, 1, and 0.1) using QIAamp viral RNA mini kit (Qiagen, Hilden, Germany) as per manufacturer’s instructions. Prior spiking, laboratory-reared mosquito homogenate was screened by RT-PCR and mRT-PCR using specific sets of primers designed for DENV and CHIKV. Spiking of RNA of DENV serotypes 1,2, 3, and 4, CHIKV, Japanese encephalitis virus (JEV), and West Nile virus (WNV) was carried out by adding an equal volume at a ratio (1:1) of virus lysate (AVL buffer) and MTH (laboratory-reared whole adult *A. albopictus* mosquitoes were macerated by mortar and pestle and then made into a 20% suspension with PBS followed by centrifugation at 5000 rpm for 30 min and collection of supernatant as a source of vector RNA) which were mixed well followed by extraction of viral RNA from the MTH. Following spiking, all samples were processed individually for RNA extraction. The RNA was eluted from the QIAspin columns in a final volume of 60 µL with elution buffer and used immediately for mRT-PCR without storage of the extracted viral RNA at −20 °C.

### 2.8. RT-PCR Reaction

For a total 50.0 µL reaction volume, a mixture of 5.0 µL of 10× LA PCR buffer, 4.0 µL of 2.5 mM MgCl_2_, 2.0 µL of 10 mM dNTP Mix, 2.0 µL of prime RNase Inhibitor, 0.2 µL/2units of *LA-Taq,* 0.2 µL/2 units of *AMV-RT*, type specific primers of 0.15 µL (sense primer of 15 nmol) and 0.4 µL of 40 nmol antisense primer), 4 µL of RNA template of virus titer (0.1-100 FFU) and 33.25 to 30.0 µL of diethyl pyrocarbonate (DEPC) treated water were used for screening the viral RNA by conventional RT-PCR.

### 2.9. mRT-PCR Reaction

For a total 50.0 µL final reaction volume, a mixture of 5.0 µL of 10× LA PCR buffer, 4.0 µL of 2.5 mM MgCl_2_, 2.0 µL of 10 mM dNTP Mix, 2.0 µL of prime RNase Inhibitor, 0.2µL/2 units of *LA Taq*, 0.2 µL/2 units of *AMV-RT*), primers, dengue consensus sense primer DCS1, 0.15 µL (15 nmol) and type specific complementary primer for each dengue serotype, D1C, 0.2 µL (20 nmol); D2 antisense primer, 0.15 µL (15 nmol), D3 antisense primer, 0.15 µL (15 nmol), D4 antisense primer, 0.20 µL (20 nmol), CHIKV-, 0.4 µL (40 nmol) Sense, CHIKV 0.4 µL (40 nmol antisense primer), and RNA template 2 µL for each virus (from titer 100.0-0.1 FFU) and 33.25 to 30.0 µL of DEPC water for the mRT-PCR reaction.

### 2.10. Optimization of Thermal Profile to Increase the Specificity and Sensitivity of the mRT-PCR

The primers used for the mRT-PCR have different melting profiles. Primer concentrations used for the mRT-PCR varied from 15 to 40 nmol, respectively, depending on the serotype and strain variation of the viruses. The thermal profile followed for cDNA synthesis with the primers mixture of mRT-PCR was 54 °C for 1 h and for PCR, 94 °C (2 min), 94 °C (30 s), 56 °C (1 min), 60 °C (2 min) for final extension 60 °C (10 min). DNA amplification required 25 cycles in the mRT-PCR assay. The DNA amplicon of each virus strain was detected using 2% agarose NA gel electrophoresis (Amersham Biosciences AB, Uppsala, Sweden), the gel was stained with ethidium bromide/MIDORI Green DIRECT) at a concentration of (10 µL/100 mL of gel)/ 1:10 ratio and the electrophoresis was conducted in TAE (Wako, Japan) buffer and the gel was visualized by UV transilluminator.

### 2.11. Statistical Analysis

The SPSS (IBM SPSS-25.0, USA) software was used to assess the significance of specificity and sensitivity of the two enzyme-based six combinations of the enzymes (reverse transcriptase and DNA polymerase) used for the mRT-PCR.

## 3. Results

### 3.1. Comparing Specificity and Sensitivity of the Primers and Enzymes Designed for mRT-PCR

Six combinations of the three DNA polymerases (LA-Taq, rTaq, and Tth) and two reverse transcriptases (RT-Ace and AMV-RT) were used to select best combinations of DNA polymerase and reverse transcriptase for increasing specificity and sensitivity of the primers designed for the single-tube, single-step mRT-PCR reaction (Table 2).

**Figure 1:**
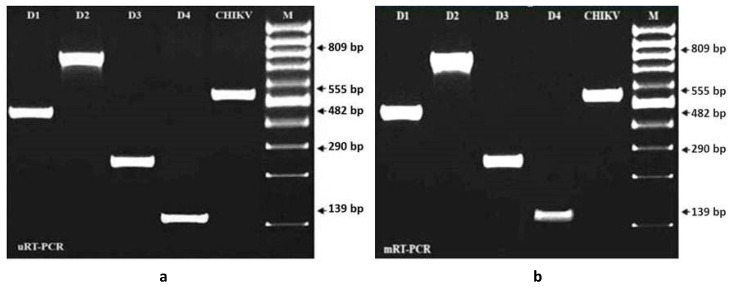
Electrophoresis of 2% agarose gel loaded with 10 µL of the PCR product, showing the specificity of the primers and enzyme for PCR products in 2% NA agarose gel electrophoresis. (**a**) Electrophoresis of 2% agarose gel loaded with 10 µL of the PCR product, showing the specificity of the primers and enzyme for the uniplex RT-PCR (uRT-PCR) products in 2% NA agarose gel electrophoresis. Lane 1, DENV-1; Lane 2, DENV-2; Lane 3, DENV-3; Lane 4, DENV-4; Lane 5, CHIKV; M is a 100bp DNA marker. (**b**) Electrophoresis of 2% agarose gel loaded with 10 µL of PCR product showing the specificity of the primers and enzyme for the mRT-PCR products in 2% NA agarose gel electrophoresis. Lane 1, DENV-1; Lane 2, DENV-2; Lane 3, DENV-3; Lane 4, DENV-4; Lane 5, CHIKV; M is a 100 bp DNA marker. The primers designed for serotype detection of DEN -2 and -4 used for uRT-PCR and mRT-PCR revealed equal degrees of specificity for serotype detection of DENV and CHIKV. One-thirtieth of the extracted RNA was amplified by both uRT-PCR and mRT-PCR. Of the enzyme combinations, LA *Taq* DNA polymerase and AMV-RT were more significant than other combinations for both the uRT-PCR and mRT-PCR. The best results were obtained by using one-third of the number of enzymes as recommended by the manufacturer.

**Figure 2:**
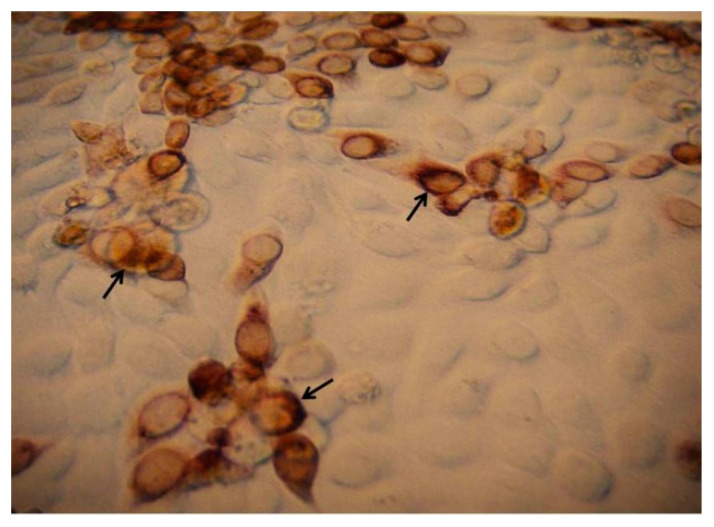
Stereomicrograph of a brown cluster of BHK-21 cells showing single focus development by DENV-infected cells 48 h post-infection (1000×).

In this study, a single-tube, single-step uRT-PCR protocol was developed to reverse transcribe the viral RNA of all four DENV serotypes and the CHIKV serotype in order to amplify the selective gene of each virus by the yield of virus-specific DNA products. Before being tested the specificity and sensitivity of the primer-sets designed for the mRT-PCR, a single pair of the primers (consensus sense and complementary for each serotype of dengue) and sense and complementary primers for CHIKV viruses were tested by uRT-PCR in this study. The primer-pairs designed for the mRT-PCR revealed equally specific and sensitive to the uRT-PCR (Figure 1 and Figure 2). The sensitivity and specificity of the enzyme combinations LA-Taq DNA polymerase and AMV-RT were significant at *p* < 0.01 and *p*< 0.05 by Duncan’s Multiple Range Test (DMRT) as compared to those of other combinations.

The DENV infected cluster of BHK-21 cells showing brown color focus as shown in Figure 2.

### 3.2. Evaluation of Specificity and Sensitivity of mRT-PCR for the Detection of Dengue and Chikungunya Viruses

**Figure 3:**
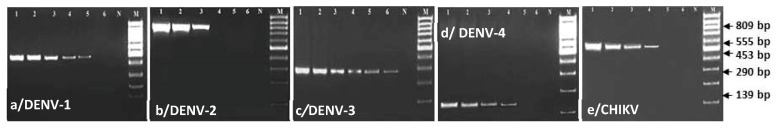
Electrophoresis of 2% agarose gel loaded with 10 µL of the PCR product, showing primer specificity and selectivity using the enzyme combination (LA-Taq+ AMV-RT) designed for the mRT-PCR. In each panel: Lane 1, 100 FFU; Lane 2, 50 FFU; Lane 3, 20 FFU; Lane 4, 10 FFU; Lane 5, 1 FFU; Lane 6, 0.1 FFU, N is a negative control and M is a 100 bp DNA marker. (**a**) Detection of DENV-1 (**b**) Detection of DENV-2 (**c**) Detection of DENV-3 (**d**) Detection of DENV-4 (**e**) Detection of CHIKV.

Using mRT-PCR, the limit of genome detection for DENV serotypes 1, 2, 3 and 4 was 1, 20, 0.1, and 10 FFU, respectively (Figure 3). The limit of genome detection for CHIKV was 10 FFU (Figure 3e).

### 3.3. Evaluation of Specificity and Sensitivity of mRT-PCR for the Detection of Dengue and Chikungunya Viruses Spiked with A. aegypti Tissue Homogenate

**Figure 4:**
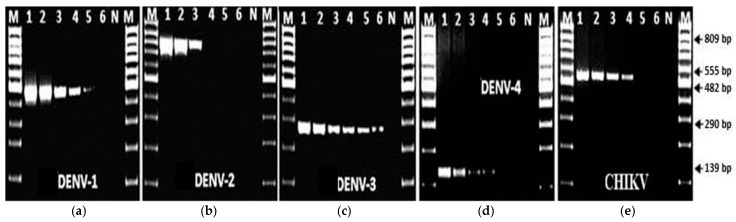
Electrophoresis of 2% agarose gel loaded with 10 µL of the PCR product, showing primer sensitivity and specificity using the enzyme combination (LA-Taq + AMV-RT) designed for the mRT-PCR. The RNA of DENV-1 (Panel a), DENV-2 (Panel b), DENV-3 (Panel c), or DENV-4 (Panel d) was spiked with mosquito tissue homogenate. The RNA of CHIKV, JEV, and WNV was spiked with mosquito tissue homogenate (Panel e). In each panel: Lane 1, 100 FFU; Lane 2, 50 FFU; Lane 3, 20 FFU; Lane 4, 10 FFU; Lane 5, 1 FFU; Lane 6, 0.1 FFU, N is a negative control and M is a 100 bp DNA marker (**a**) Detection of DENV-1 (**b**) Detection of DENV-2 (**c**) Detection of DENV-3 (**d**) Detection of DENV-4. (**e**) Detection of CHIKV.

In order to assess the applicability of the mRT-PCR for detection and differentiation of the spiked viruses with vector mosquito homogenates, a known concentration (100, 50, 20, 10, 1 and 0.1 FFU) of each of the four serotypes of dengue (DEN 1–4) and CHIKV, JEV, and WN viruses were spiked with ten times higher RNA of the specific mosquito vector (*A. aegypti*) cell homogenate (1 µg of viral RNA added with 10 µg of vector tissue RNA. Each of the spiked viral RNA template was used for mRT-PCR (Figure 4).

The sensitivity and specificity of the mRT-PCR for the detection of DENV and CHIKV genome using specific primer sets designed for the mRT-PCR did not show cross-reactivity with mosquito cell-derived RNA and RNA of other viruses of the family *Flaviviridae* (Figure 4). The mRT-PCR was repeated three times for each test isolate of dengue (DENV-1, DENV-2, DENV-3, and DENV-4) and CHIKV viruses to ensure reproducibility of results on the sensitivity and specificity of the primer sets designed for serotype detection of DENV and CHIKV (Figure 4a–e).

### 3.4. Comparing Specificity and Sensitivity of mRT-PCR and Virus Isolation and Validation of the Test for Serotyping of DENV and CHIKV Obtained from Clinical Samples

A total of 650 serum samples of dengue-suspected febrile patients were tested for DENV and CHIKV by mRT-PCR and virus isolation. It was found that 31.7% (206/650) samples were positive for DENV and 22.9% (149/650) were positive for CHIKV using mRT-PCR (Table 3). On the contrary, only 45 (23.16%) samples were positive for DENV using virus isolation in C6/36 cells (Table 3). The increased number of CHIKV-positive samples in 2017 may be explained by the increased number of mosquito vectors [46]. We did not detect any co-infection with more than one serotype of DENV or with both DENV and CHIKV.

## 4. Discussion

Among viral diseases, dengue and chikungunya viral fever are the leading causes of human death [3,16,47]. Early and rapid diagnosis using antibody detection methods, nucleic acid detection methods or virus isolation is crucial for the development of appropriate control measures.

Lanciotti et al. developed a group-specific mRT-PCR for the detection and differentiation of the four serotypes of DENV using a single-tube mRT-PCR reaction in which the sensitivity and specificity were less than the type-specific uRT-PCR. [33]. In the present study, a novel single-tube, single-step mRT-PCR was developed for the rapid detection of DENV serotypes and the differentiation of DENV from CHIKV. The assay is rapid, highly specific, highly sensitive, and cost-effective, and easily operated.

To increase the sensitivity and specificity of the newly developed mRT-PCR assay, several changes were made to the previously used protocol. Since the primary objective was to develop a mRT-PCR that could detect viral RNA of the serotypes of DENV and CHIKV in a single-tube, single-step reaction, two complementary primers specific for DENV-2 and DENV-4 were designed in addition to a pair of sense and complementary primers specific for the CHIKV. In a study by Harris et al., both the sense and complementary primers of CHIKV revealed several annealing sites in the genome sequences of the DENV-2 and DENV-4 serotypes when used in the mRT-PCR [36]. Along with the replacement of the primers, the cDNA synthesis reaction temperature (50 °C) used at the reverse transcription step was increased to 60 min. The amplification cycles of the two-enzyme reaction systems were also reduced from 40 cycles to 25 cycles in order to get more specific PCR products of each of the five viruses in the shortest possible time. It was also observed that changing the primers, amplification cycles, and temperature at the reverse transcription step were not beneficial unless and until the correct combination and concentrations of the two enzymes (DNA polymerase and reverse transcriptase) were used.

Among the six different combinations of the two-enzyme protocol systems used in the mRT-PCR, the combination of LA Taq DNA polymerase and AMV-RT showed the best results when compared with the other five combinations (Table 2). Therefore, replacement of the two complementary primers for DENV 2 and DENV 4, reduction of PCR amplification cycles, increasing the temperature at the reverse transcription step, and the enzyme combination might have played a significant role in increasing the specificity and sensitivity of the mRT-PCR. The sensitivity and specificity of the mRT-PCR designed for the detection and differentiation of all four serotypes of DENV and CHIKV were different from results of the mRT-PCR used for the detection of all four serotypes of DENV alone [36]. The reduced sensitivity and specificity of the mRT-PCR assays previously reported by Harris et al. and by Kumaria and Chakravarti [36,43] for genome detection of DENV might be explained by the use of higher concentration (0.5–1.0 µM) of the primers for each of the four DENV serotypes, the use of low temperature (42 °C) at the reverse transcription step, and the number of cycles (40) of the RT-PCR reaction.

Primer design and concentrations were found to be the key factors for the sensitivity and specificity of the assay, and to check for the presence of annealing sites of the sense and complementary primers of DENV serotypes. Since the current mRT-PCR is designed for the genome detection of more than one virus in a single-tube reaction, it is important to adjust the concentration of all the primers. Higher concentrations of primers may result in the formation of a complex in the reaction mixture of mRT-PCR. Efficient viral RNA extraction methods are required for the accurate detection of viruses either from infected mosquito vectors or from the spiked mosquito vector homogenates in order to avoid RNA degradation or PCR inhibition. The efficiency of RNA extraction from the spiked viruses used in the present study using the QIAamp viral RNA mini extraction kit (Qiagen, Hilden, Germany) indicated that this method could be successfully used for the extraction of viral RNA without contamination with RNA of mosquito vectors collected from the field. Viral RNA extracted using the QIAamp mini extraction kit in the present study showed good sensitivity without any inhibition in amplification for either the uRT-PCR or mRT-PCR. This was similar to observations from a previous report [37].

Virus isolation by cell culture using the *A. albopictus* C6/36 cell line is considered a gold standard for the detection of dengue and other viruses of the family *Flaviviridae* and *Togaviridae* [33,43]. In this study, a total of 650 acute-phase febrile patient serum samples were collected and were subjected to serotype detection of DENV and CHIKV by mRT-PCR, virus isolation, and NS1 Ag detection kit for dengue. Of the 650 samples, 250 (41.6%) were DENV-positive and 135 (22.5%) were CHIKV positive by the mRT-PCR, whereas only 45 (6.9%) samples were positive for virus isolation (DENV-2 and DENV-3) by cell culture and 175 (26%) were positive by NS1 rapid Ag detection. Therefore, the sensitivity of the mRT-PCR developed in this study was higher than that of virus isolation from the clinical samples, as was previously reported [36,48]. The reagents and equipment used for isolation and detection of viruses from field samples using mosquito cell line are expensive. In addition, the procedures are very laborious and time consuming as compared to the mRT-PCR. The detection of viruses and their serotyping by the mRT-PCR method does not have such limitations and the viral RNA could be easily obtained even if the live viruses are not viable in the samples. Using mRT-PCR, a large number of samples can be processed, and viral genome detection could be completed in a short time. The mRT-PCR test was developed with the objective of nucleic acid detection of DENV and CHIKV in the active phase of febrile infection. The mRT-PCR methods are easier to perform and more cost-effective (cost is estimated at 10 cents/ sample) and can be performed at any laboratory by technicians with regular experience in RT-PCR/PCR. On the other hand, RT-LAMP and RT-RPA, which are highly sophisticated, expensive (more than 2 $/ sample), and require more trained personnel.

The enzyme combinations (AMV-RT reverse transcriptase and LA-Taq DNA polymerase), primer selection, primer concentration, enzyme concentration, and thermal profile designed for the mRT-PCR increased the specificity and sensitivity of the RT-PCR for the simultaneous detection of the serotypes of DENV and CHIKV from any sample (patient sera, ICF, and mosquitos). The sensitivity and specificity of the single-tube, single-step mRT-PCR were verified using reference viruses of the four serotypes of DENV and CHIKV, which indicated that this assay could be used for rapid detection of the serotypes of DENV and CHIKV simultaneously from clinical and field samples.

The newly developed single-tube, single-step mRT-PCR can rapidly detect and differentiate all four DENV serotypes from CHIKV from clinical, laboratory, and mosquito samples without showing artifacts. This assay can be used as a tool for routine diagnosis of DENV and CHIKV due to its speed, sensitivity, specificity, and cost-effectiveness.

## Figures and Tables

**Table 1:** List of primers designed for the detection of DENV and CHIKV by mRT-PCR.

Primers	Sequences (5′-3′)	Nucleotide Position	Orientation	Accession No.	Strain Name
* DC-S	TCAATATGCTGAAACGCGCGAGAAACCG	134–161	Sense	M84727	DENV-2 16681
* D1-C	CGTCTCAGTGATCCGGGGG	586–568	Complementary	M23027	DENV-1
D2-C	ACGCATTGTCATTGAGGGAG	942–923	Complementary	AF022434	DENV-2 ThNH-7/93
* D3-C	TAACATCATCATGAGACAGAGC	421–400	Complementary	L11423	DENV-3 H-87
D4-C	GGAAAGGACTCGCAAAAAC	272–254	Complementary	M14931	DENV-4
CHIKV-S	TACAGCACACAGCACCAT	10,745–10,762	Sense	AF369024	CHIKV S27-African prototype
CHIKV-C	ACGCATAGCACCACGATTA	11,294–11,276	Complementary		

* Selected from the primer lists of previously published articles [33,36].

**Table 2:** Sensitivity of the mRT-PCR for serotype detection of DENV and CHIKV.

DNA Polymerase	Reverse Transcriptase	Serotype Detection Limit of the mRT-PCR				
		DENV-1	DENV-2	DENV-3	DENV-4	CHIKV
		FFU Mean ± SE	FFU Mean ± SE	FFU Mean ± SE	FFU Mean ± SE	FFU Mean ± SE
*LA-Taq+*	*AMV-RT*	1.00 ± 0.00c	20.00 ± 2.89b	0.10 ± 0.00c	10.00 ± 0.00b	10.00 ± 0.00b
*r-Taq+*	*AMV-RT*	10.00 ± 2.89b	20.00 ± 2.89b	1.00 ± 0.00b	20.00 ± 0.00a	20.00 ± 2.89a
*Tth+*	*AMV-RT*	10.00 ± 2.89b	50.00 ± 5.77a	1.00 ± 0.00b	20.00 ± 2.89a	20.00 ± 2.89a
*LA-Taq+*	*RT-ACE*	20.00 ± 2.89a	50.00 ± 2.89a	1.00 ± 0.00b	20.00 ± 0.00a	20.00 ± 0.00a
*r-Taq+*	*RT-ACE*	20.00 ± 2.89a	50.00 ± 1.15a	10.00 ± 2.89a	20.00 ± 2.89a	20.00 ± 2.89a
*Tth+*	*RT-ACE*	20.00 ± 2.89a	50.00 ± 2.89a	10.00 ± 1.15a	20.00 ± 2.89a	20.00 ± 0.00a
*p* value		0.001	0.001	0.001	0.023	0.023
Level of sig.		**	**	**	*	*

** Significant at *p* < 0.01 * Significant at *p* < 0.05.

**Table 3:** Validation of mRT-PCR for the detection of DENV and CHIKV and comparison of its sensitivity with virus isolation in C6/36 cells and NS1 Ag detection methods.

Country	Sampling Years	No. of Samples	No. of Positive Samples Using mRT-PCR					Total No. and % of Positive Samples		No. of Positive Samples Using Virus Isolation			Total No. and % of Positive Samples Using Virus Isolation		Total No. and % of Positive Samples Using Dengue NS1 Ag Kit	
			D1	D2	D3	D4	CHIKV	DENV	CHIKV	D1	D4	CHIKV	DENV	CHIKV	DENV	CHIKV
**Bangladesh**	2016	200	0	10	90	0	5	100 (50%)	5 (2.5%)	0	0	0	25 (12.5%)	0	85 (42%)	0
	2017	300	0	1	35	0	140	36 (12%)	140 (46.7%)	0	0	0	8 (2.7%)	0	30 (10%)	0
	2018	150	0	0	70	0	4	70 (46.7%)	4 (2.7%)	0	0	0	12 (8%)	0	60 (40%)	0
**Total**	3 years	650	0	11	195	0	149	206 (31.7%)	149 (22.9%)	0	0	0	45 (23.2%)	0	175 (92%)	0
